# Dexamethasone Causes Hypertension in Rats Even Under Chemical Blockade of Peripheral Sympathetic Nerves

**DOI:** 10.3389/fnins.2019.01305

**Published:** 2019-12-06

**Authors:** Alexandra E. Soto-Piña, Cynthia Franklin, C. S. Sheela Rani, Elizabeth Fernandez, Elías Cardoso-Peña, Alejandra D. Benítez-Arciniega, Helmut Gottlieb, Carmen Hinojosa-Laborde, Randy Strong

**Affiliations:** ^1^Facultad de Medicina, Universidad Autónoma del Estado de México, Toluca, Mexico; ^2^Feik School of Pharmacy, University of the Incarnate Word, San Antonio, TX, United States; ^3^Department of Pharmacology, The University of Texas Health Science Center at San Antonio, San Antonio, TX, United States; ^4^Audie L. Murphy Division, South Texas Veterans Health Care System, San Antonio, TX, United States; ^5^Unidad de Medicina Familiar 220, Instituto Mexicano del Seguro Social, Mexico City, Mexico

**Keywords:** dexamethasone, hypertension, adrenal medulla, 6-OHDA, renal denervation, sympathetic nerves

## Abstract

Synthetic glucocorticoids (GCs) are widely used to treat inflammatory conditions. However, chronic use of GCs can lead to hypertension. The cause of this undesired side effect remains unclear. Previously, we developed an *in vivo* rat model to study the mechanisms underlying hypertension induced by the chronic administration of the potent synthetic GC, dexamethasone (DEX) and found that the catecholamine biosynthetic pathway plays an important role. In the current study, we used this model to investigate the role of the adrenal medulla, renal nerves, and other peripheral sympathetic nerves in DEX-induced hypertension. After 5 days of baseline telemetric recording of mean arterial pressure (MAP) and heart rate (HR), rats were subjected to one of the following treatments: renal denervation (RDNX), adrenal medullectomy (ADMX), 6-hydroxydopamine (6-OHDA, 20 mg/kg, i.p.) to induce chemical sympathectomy, or a combination of ADMX and 6-OHDA. On day 11, the animals received vehicle (VEH) or DEX in drinking water for 7 days, with the latter causing an increase in MAP in control animals. ADMX and RDNX by themselves exacerbated the pressor effect of DEX. In the chemical sympathectomy group, DEX still caused a rise in MAP but the response was lower (ΔMAP of 6-OHDA/DEX < VEH/DEX, *p* = 0.039). However, when ΔMAP was normalized to day 10, 6-OHDA + DEX did not show any difference from VEH + DEX, certainly not an increase as observed in DEX + ADMX or RDNX groups. This indicates that sympathetic nerves do not modulate the pressor effect of DEX. TH mRNA levels increased in the adrenal medulla in both VEH/DEX (*p* = 0.009) and 6-OHDA/DEX (*p* = 0.031) groups. In the 6-OHDA group, DEX also increased plasma levels of norepinephrine (NE) (*p* = 0.016). Our results suggest that the activation of catecholamine synthetic pathway could be involved in the pressor response to DEX in animals even under chemical sympathectomy with 6-OHDA.

## Introduction

Hypertension is a disease that has been linked to central nervous system alterations, elevated sympathetic activity, and inflammatory damage to end organs and arteries (McMaster et al., 2015; Tomiyama et al., 2017). Synthetic glucocorticoids (GCs) are anti-inflammatory drugs that produce hypertension in patients undergoing chronic GC treatment (Panoulas et al., 2008). The mechanisms by which synthetic GCs produce hypertension are not well known. Dexamethasone (DEX) is a potent synthetic GC that does not readily cross the brain blood barrier (De Kloet, 1997). This suggests that DEX may produce hypertension through a peripheral mode of action. Classically, it is believed that the pressor effect of endogenous or synthetic GCs, mediated by the GC receptor (GR), is associated with renal sodium and water imbalance (Whitworth et al., 2000; Mangos et al., 2003). It is plausible that synthetic GC-induced hypertension is mediated by the activation of the renal nerves, since it augments sodium and water reabsorption, elevates renal vascular resistance, and contributes to the onset and maintenance of hypertension (DiBona, 2002; DiBona and Esler, 2010). Moreover, peripheral administration of the endogenous GC, corticosterone, increases baseline mean arterial pressure (MAP) and shifts the baroreflex curve midpoint to the right for renal sympathetic nerve activity (Scheuer and Mifflin, 2001). However, the hypothesis that GC effects are mediated via renal mechanisms is questionable because the homozygous deletion of the GR gene in the distal nephron does not prevent DEX-induced hypertension (Goodwin et al., 2010). As an alternative, we hypothesize that the adrenal medulla, which is a major source of plasma catecholamines, plays a role in DEX-induced hypertension. We recently showed that chronic DEX administration, in addition to increasing MAP, induces the transcription of tyrosine hydroxylase (TH), the rate limiting enzyme in catecholamine synthesis, in the rat adrenal medulla (Soto-Piña et al., 2016). At the molecular level, the mechanism of DEX regulation of TH transcription through the GR involves an atypical GC-responsive element in the TH promoter region (Rani et al., 2009, 2013).

The development of therapeutic treatments for hypertension has included the use of pharmacological and surgical ablations which are also useful approaches for identifying the role of nerves and sympathetic ganglia. For instance, renal denervation (RDNX) is useful in the treatment of human drug-resistant hypertension (Krum et al., 2009; Schlaich et al., 2011) and in several experimental animal models of hypertension (Katholi et al., 1984; Norman et al., 1984; O’Hagan et al., 1990; Ojeda et al., 2007; Dagan et al., 2008). Furthermore, adrenal medullectomy (ADMX) is effective in diminishing hypertension in models of deoxycorticosterone-salt and spontaneously hypertensive rats (SHRs) (Sakaguchi et al., 1983; Lange et al., 1998).

Sympathetic nerves are known to be essential in the generation of hypertension induced by endogenous GCs and mineralocorticoids, but whether hypertension induced by synthetic GCs requires sympathetic nerves is unknown. Chemical sympathectomy with 6-hydroxydopamine (6-OHDA) induces selective destruction of sympathetic nerve terminals by accumulation of the neurotoxin specifically via noradrenergic transporters, causing a depletion of norepinephrine (NE) and reduced TH activity (Thoenen and Tranzer, 1968; Tranzer and Thoenen, 1968). The advantage of chemical sympathectomy with the 6-OHDA neurotoxin is twofold: firstly, its effects are specific to the periphery since it does not cross the brain blood barrier (Fink and Mirkin, 1991), and secondly, the adrenal medulla is spared the catecholamine depletion by this neurotoxin (Stachowiak et al., 1986), due to the absence of NE transporters on noradrenergic chromaffin cells. Thus, systemic 6-OHDA administration causes a systemic peripheral sympathectomy that also reduces hypertension in animal models (Chalmers et al., 1984; Wu et al., 1998). Therefore, chemical sympathectomy with 6-OHDA is a useful approach to study the role of sympathetic nerves in animal models of hypertension.

To date the role of the peripheral sympathetic nervous system in hypertension induced by synthetic GCs under chronic conditions is unknown. In a previous study, the administration of the TH inhibitor, alpha-methyl-para-tyrosine (αMPT), reduced the pressor effect of DEX in a model of hypertension induced by this GC (Soto-Piña et al., 2016). However, it was not clear whether the influence was on the adrenal medulla and/or sympathetic nerves. Therefore, the hypothesis of this study is that the renal nerves, adrenal medulla, and systemic peripheral sympathetic nerves are potential contributors to DEX-induced hypertension. The goal of the study was to determine whether ADMX, RDNX, sympathectomy by 6-OHDA, or a combination of ADMX and 6-OHDA can reveal the contribution of the sympathetic nervous system with the pressor effect of DEX. To do that we used a novel model of DEX-induced hypertension in which the elevation of blood pressure was induced by oral chronic administration of DEX to rats that were implanted with radiotelemetry, to enable continuous monitoring of blood pressure and heart rate (HR).

## Materials and Methods

### Animals

Male Fisher 344 rats were purchased from Envigo (Madison, WI, United States), 12–14 weeks old, and 300–310 g in weight. Male rats were included in the study because their body weight and TH expression is stable as they age (Tumer and Larochelle, 1995) and to prevent blood pressure alterations related to estrogen signaling and age-related variations. They were maintained on a 14/10-h light/dark cycle (lights on at 7 AM and off at 9 PM). Water and standard chow (Teklad #7012, Envigo, Madison, WI, United States) were provided *ad libitum* throughout the whole study. At their arrival, rats stayed 36 h in a quarantine room, to prevent any cross contamination. After that, they were transferred to the housing telemetry room. They stayed there for 5 days for further acclimation and were under observation during this period to identify any signs of stress. Body weight and water intake were measured throughout the experimental treatments. All experiments were approved and performed under the guidelines of the Institutional Animal Care and Use Committee (IACUC) of the University of Texas Health Science Center at San Antonio.

### Radiotelemetry Implantation and Data Collection

The radiotelemetry implant was performed as before (Soto-Piña et al., 2016) using an aseptic procedure. After the 5-day acclimation period, rats underwent inhaled anesthesia with (2% isoflurane in oxygen), the abdominal aorta was exposed and two clamps were made to prevent bleeding, one underneath the renal artery and another above the iliac arteries. A small perforation on the aorta was made using a sterilized needle at the level of the mesenteric artery, to introduce the telemetry catheter (CA11PA-C40, Data Science International, St. Paul, MN, United States). The abdominal wound was sutured and the animals were placed individually in cages with UV sterilized bedding, and they were given 20% infant Ibuprofen in drinking water to ameliorate surgery pain for 3 days. Animals had a recovery period of 8 days before turning on the telemeters. At the end of this period, baseline for MAP and HR were continuously recorded at 10-min intervals per hour, for 5 days. Data were collected using Dataquest A.R.T. 4.1 software (Data Science International, St. Paul, MN, United States). Since diurnal MAP and HR responded similarly to the different experimental treatments, we report both of them during the light phase (7:00 AM–9:00 PM). In the experimental time line, day 1 indicates the first day of recording to establish the baseline and the numeration continues until the last day of recording/treatment on day 17. Data for MAP and HR are reported as the change (Δ) from baseline. This was computed as follows: the average of daily MAP and HR was obtained considering the six measures per hour during the light phase (14 h period). Then the mean average of the 5 days of baseline was subtracted from the mean average of each day of recording for MAP and HR, respectively (see Supplementary Tables S1, S2). Data are shown in graphs as the group median of daily ΔMAP and ΔHR. The mean, confidence interval, and limits of the median (rank) are depicted in Supplementary Files S1–S5.

Normalized ΔMAP and ΔHR were obtained by subtracting the mean average on day 10 from the daily average mean of ΔMAP or ΔHR on days 10–17. This was performed for each animal, then, the median, confidence interval, as well as upper and lower limits were computed for each group treated with DEX. Finally, activity (considered as the horizontal movement inside the cage) was measured with telemetry as well, it was expressed as counts/min.

### Renal Denervation

A group of rats was used to determine the influence of the renal nerves on DEX-induced hypertension. Animals underwent bilateral RDNX after 5 days of baseline recordings (day 6) under isoflurane anesthesia. Through flank incisions, the left and right kidneys were exposed and the renal arteries and veins were stripped of any nerve bundles visible using a 25× stereo microscope. To increase the efficiency of the RDNX, each renal artery and renal vein were brushed with 20% phenol/ethanol. After surgery, rats were placed in individual cages. In rats receiving SHAM surgery, only flank incisions were performed. MAP and HR responses to RDNX were recorded for 4 days.

### Adrenal Medullectomy

Bilateral flank incisions were performed to expose the right and left adrenal glands under isoflurane anesthesia and aseptic conditions. This procedure was performed after baseline telemetric recording on day 6 as in the RDNX experiment. A small incision was made in the adrenal cortex and the adrenal medulla was extruded by applying gentle pressure to the adrenal gland. In animals that had SHAM surgery, a small incision was made in the adrenal cortex, but the adrenal medulla was not extruded. The adrenal gland was then repositioned over the kidney and incisions were closed. The animals were returned to their respective cages. MAP and HR were recorded for the following four post-surgery days. We validated the effect of ADMX by measuring plasma catecholamines 4 days after the surgery in separate ADMX and SHAM groups of animals without the radiotelemetry implant.

### 6-OHDA Sympathectomy and Combination With ADMX

Chemical sympathetic denervation was performed by the administration of 6-OHDA hydrobromide (Sigma Aldrich, St. Louis, MO, United States) 20 mg/kg i.p. diluted in a vehicle (VEH) of 0.9% saline and 0.1% ascorbic acid. The i.p. injections were performed for six alternative days (days 6, 8, 10, 12, 14, and 16). This dosing schedule was used to minimize 6-OHDA side effects. We had previously performed a pilot study with 50 mg/kg, but animals showed some adverse effects such as diarrhea and fatigue. Furthermore, a control group was included and it received similar injections of vehicle (saline and ascorbic acid). For the combined ADMX and 6-OHDA treatment, animals were subjected to isoflurane anesthesia and the removal of the adrenal medulla was performed as described above. Five days after ADMX surgery, animals were treated with 6-OHDA hydrobromide (Sigma Aldrich, St. Louis, MO, United States) 20 mg/kg i.p., on days 10, 12, 14, and 16.

### DEX Treatment

Rats received DEX 21-phosphate disodium salt (Sigma Aldrich, St. Louis, MO, United States) at 0.03 mg/kg/day in drinking water from days 11 to 17 of the study, regardless of the surgical or chemical procedure performed previously. Control groups received only drinking water (VEH).

### Measurement of Plasma and Renal Catecholamines by High-Performance Liquid Chromatography

At the end of the experiment (day 17), animals were euthanized under isoflurane anesthesia and blood was collected from the vena cava using heparinized syringes. Samples were centrifuged at 4000 × *g* for 15 min within an hour and plasma was separated and stored at −80°C until the assay. Plasma EPI and NE were isolated using an alumina adsorption–extraction kit (catalog # 45-0141 ESA Biosciences Inc., Chelmsford, MA, United States). Catecholamine extracts (replicates of 50 μL) were processed by High-Performance Liquid Chromatography (HPLC) with electrochemical detection (ESA Biosciences Inc., Chelmsford, MA, United States). To measure renal cortex catecholamines, tissue samples were homogenized in 0.1 M perchloric acid and analyzed by HPLC as previously described (Wey et al., 2012).

### TH mRNA Content in the Adrenal Medulla

The adrenal medullae were collected and frozen on dried ice after euthanasia. Total RNA was isolated using a commercial kit (Absolutely RNA, Stratatage) according to manufacturer instructions. The expression of TH was assessed by two-step qPCR. The quality of total RNA was analyzed in 2% agarose gel, and cDNA was synthesized using a High-Performance cDNA Reverse Transcription kit (Thermo Fisher Scientific, Inc., United States). Sample reactions were set up in the presence of the MMLV-RT reverse transcriptase (+RT) and without the enzyme (−RT) as control. A non-template control was included as well. To measure TH gene expression in the adrenal medulla, we performed a multiplex qPCR rat TH qPCR assay having the best coverage with the primer and probe sequences spanning exons 12–13 (Rn00562500_m1, Thermo Fisher Scientific, Inc., United States), and for housekeeping gene, used eukaryotic 18S RNA labeled with VIC-MGB_PL (Hs99999901_s1, Thermo Fisher Scientific, Inc., United States). The reactions were set in a multiplex assay with a Taqman master reaction mix. The qPCR was performed using a 7900 Fast Real-Time cycler (Applied Biosystems, Inc.). Sample analysis was performed in duplicates. The results were obtained by the ΔΔ cycle threshold method. The relative expression of TH was calculated as the average difference in cycle threshold (Cq) of TH and 18S, between the VEH/VEH group and each testing group (VEH/DEX, 6-OHDA/VEH, and 6-OHDA/DEX).

### Statistical Analysis

ΔMAP and ΔHR data were not normally distributed using the Kolmogorov–Smirnov test; therefore, Friedman Repeated Measures Analysis of Variance on Ranks with Tukey’s multiple comparisons analysis were performed for groups comparisons. SigmaStat 4.0 statistical package (Systat Software, Inc., San Jose, CA, United States) was used to carry out these analyses. Body weight, water intake, and activity were analyzed using two-way ANOVA with Dunn’s multiple comparisons analysis. Plasma EPI and NE in the ADMX experiment were analyzed using Student’s *t*-test. One-way ANOVA analyzed differences of NE concentrations in the renal cortex with Bonferroni’s *post hoc* test. TH mRNA expression and plasma catecholamines in the 6-OHDA experiment were compared using Kruskal–Wallis test and Dunn’s multiple comparisons analysis. Data of catecholamines and TH mRNA were analyzed using Prism v8 (Graph Pad Software Inc., La Jolla, CA, United States). A value of *p* < 0.05 was considered as a significant level.

## Results

### Effect of RDNX on DEX-Induced Hypertension

**FIGURE 1:**
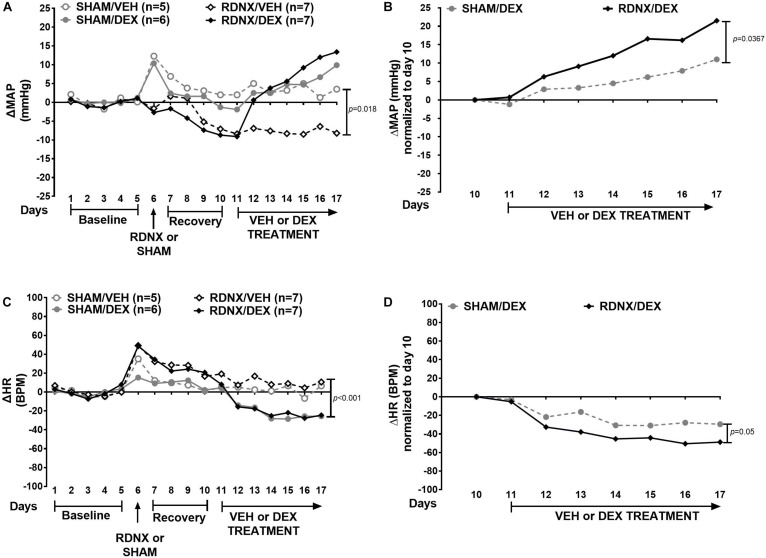
Effect of bilateral renal denervation (RDNX) on MAP and HR. Data represent the median of ΔMAP and ΔHR. Top panel shows the time course of responses in **(A)** mean arterial pressure (ΔMAP) and **(B)** ΔMAP normalized to day 10. Bottom panel shows **(C)** heart rate (ΔHR) and **(D)** ΔHR normalized to day 10. The administration of drugs and surgical procedure is indicated with arrows below the *x*-axis. Friedman Repeated Measures Analysis of Variance on Ranks with Tukey’s test were used to compare responses between groups. Confidence intervals for the median and the mean are shown in Supplementary File S1.

ΔMAP (Figure 1A) was different between groups (χ^2^ = 9.9, *p* = 0.019). Moreover, ΔMAP in the RDNX/VEH group was significantly lower than the SHAM/VEH group (*p* = 0.018). Normalized ΔMAP in the RDNX–DEX (Figure 1B) was significantly higher than the SHAM/DEX (*p* = 0.0367).

In Figure 1C, HR (Figure 1C) was significantly different between groups (χ^2^ = 14.7, *p* = 0.002). In addition, ΔHR in the RDNX/VEH was more elevated than the SHAM/DEX group (*p* = 0.001). Normalized ΔHR (Figure 1D) in RDNX/DEX group was significantly lower than the SHAM/DEX group (*p* = 0.05).

The completeness of bilateral RDNX was verified by measuring NE content in the cortex of each kidney at the end of the experiments (Table 1). There was a reduction in NE concentration in RDNX groups treated with either VEH or DEX (*p* < 0.001 vs. SHAM/VEH). Moreover, NE concentration was significantly elevated in the right renal cortex of the SHAM/DEX group (*p* < 0.05 vs. SHAM/VEH).

**TABLE 1:** NE content in renal cortex after RDNX and DEX.

	**Right renal cortex**				**Left renal cortex**			
		
**Treatment**	**SHAM**	***n***	**RDNX**	***n***	**SHAM**	***n***	**RDNX**	***n***
VEH	9.1 ± 1.7	5	0.2 ± 0.1^b^	7	8.7 ± 1.2	5	2.0 ± 0.8^b^	7
DEX (0.3 mg/kg/day)	12.1 ± 1.1^a^	6	0.1 ± 0.0^b^	7	9.5 ± 1.2	6	0.2 ± 0.1^b^	7

*Values are mean ± SEM; *n*, number of renal cortex tissue samples per group. NE concentration in renal cortex is expressed in ng/mg tissue protein; ^*a*^*p* < 0.05, ^*b*^*p* < 0.001 vs. SHAM/VEH renal cortex.*

### Effect of ADMX on DEX-Induced Hypertension

**FIGURE 2:**
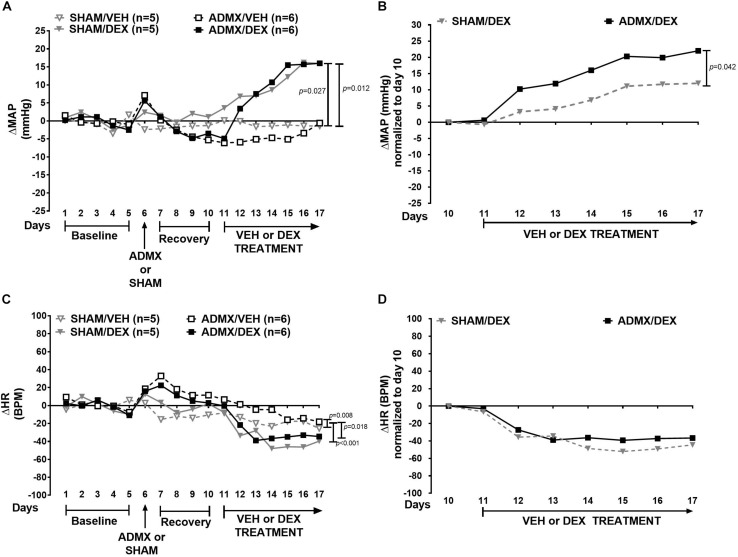
Effect of bilateral adrenal medullectomy (ADMX) on MAP and HR. Data are expressed as median. Top panel shows the time course of responses in **(A)** mean arterial pressure (ΔMAP) and **(B)** ΔMAP normalized to day 10. Bottom panel shows **(C)** heart rate (ΔHR) and **(D)** ΔHR normalized to day 10. Comparisons of responses in ΔMAP and ΔHR were performed with Friedman Repeated Measures Analysis of Variance on Ranks with Tukey’s *post hoc* test. Confidence intervals are presented in Supplementary File S2.

ΔMAP in all groups was also significantly different (χ^2^ = 12.0, *p* = 0.007). ΔMAP in SHAM/DEX was higher than in SHAM/VEH (*p* = 0.027) and ADMX/VEH (*p* = 0.012) groups (Figure 2A). Normalized ΔMAP (Figure 2B) to day 10 in the ADMX/DEX was greater than the SHAM/DEX group (*p* = 0.042). In addition, ΔHR (Figure 2C) was significantly different between groups (χ^2^ = 19.8, *p* < 0.001). ΔHR in ADMX/VEH group was higher than in SHAM/VEH (*p* = 0.008), SHAM/DEX (*p* < 0.001), and ADMXDEX (*p* = 0.018) groups. Normalized ΔHR (Figure 2D) was not significantly different between groups (*p* = 0.382).

On day 10, in a separate group of rats (without telemetry transmitters), plasma concentrations of NE and EPI in ADMX animals were 1762 ± 264 and 143 ± 44 pg/mL, respectively. These concentrations were lower than those in the SHAM groups (NE: 3627 ± 716 pg/mL, *p* = 0.020; and EPI: 593 ± 82 pg/mL, *p* = 0.005). Plasma catecholamines in ADMX rats corresponded to a 52% decrease in plasma NE and a 76% decrease in EPI.

### Effect of 6-OHDA on DEX-Induced Hypertension

**FIGURE 3:**
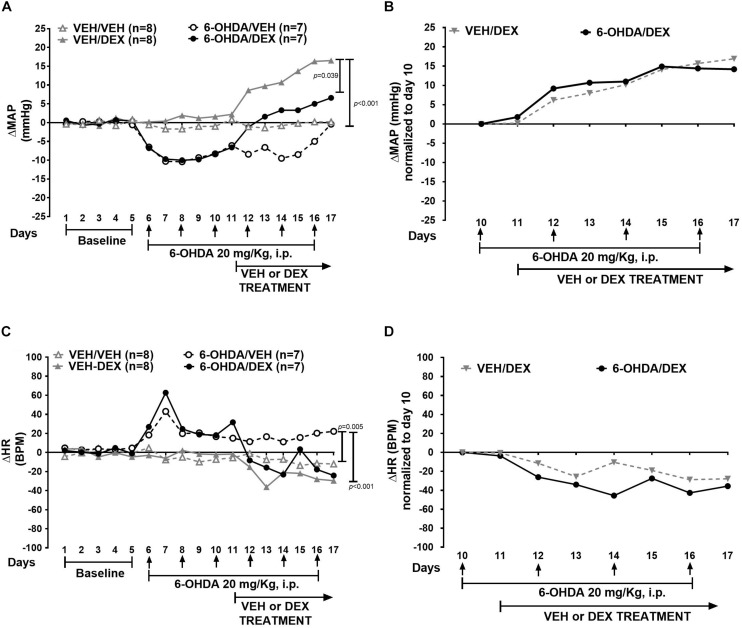
Effect of 6-day 6-OHDA administration on ΔMAP and ΔHR in DEX-induced hypertension. Data represent the median of ΔMAP and ΔHR in animals treated with VEH (gray) and 6-OHDA (black). Top panel shows **(A)** effect of 6-OHDA and DEX on ΔMAP and **(B)** ΔMAP normalized to day 10. Bottom panel shows **(C)** ΔHR and **(D)** normalized ΔHR to day 10. Comparisons were performed with Friedman Repeated Measures Analysis of Variance on Ranks with Tukey’s *post hoc* test. Confidence intervals are shown in Supplementary File S3.

In this experiment, ΔMAP was significantly different in all groups (χ^2^ = 18.7, *p* < 0.001). Furthermore, ΔMAP was higher in the VEH/DEX group than the 6-OHDA/VEH (*p* < 0.001) and 6-OHDA/DEX (*p* = 0.039) groups (Figure 3A). There was no difference (*p* = 0.422) between normalized ΔMAP (Figure 3B) between the VEH/DEX and 6-OHDA/DEX groups. ΔHR was significantly different between groups (χ^2^ = 20.9, *p* < 0.001). Moreover, ΔHR in the 6-OHDA/VEH group was higher than the VEH/VEH (*p* = 0.005) and VEH/DEX (*p* < 0.001) groups (Figure 3C). Normalized ΔHR in the VEH/DEX and 6-OHDA/DEX groups was not different (*p* = 0.069) (Figure 3D).

**FIGURE 4:**
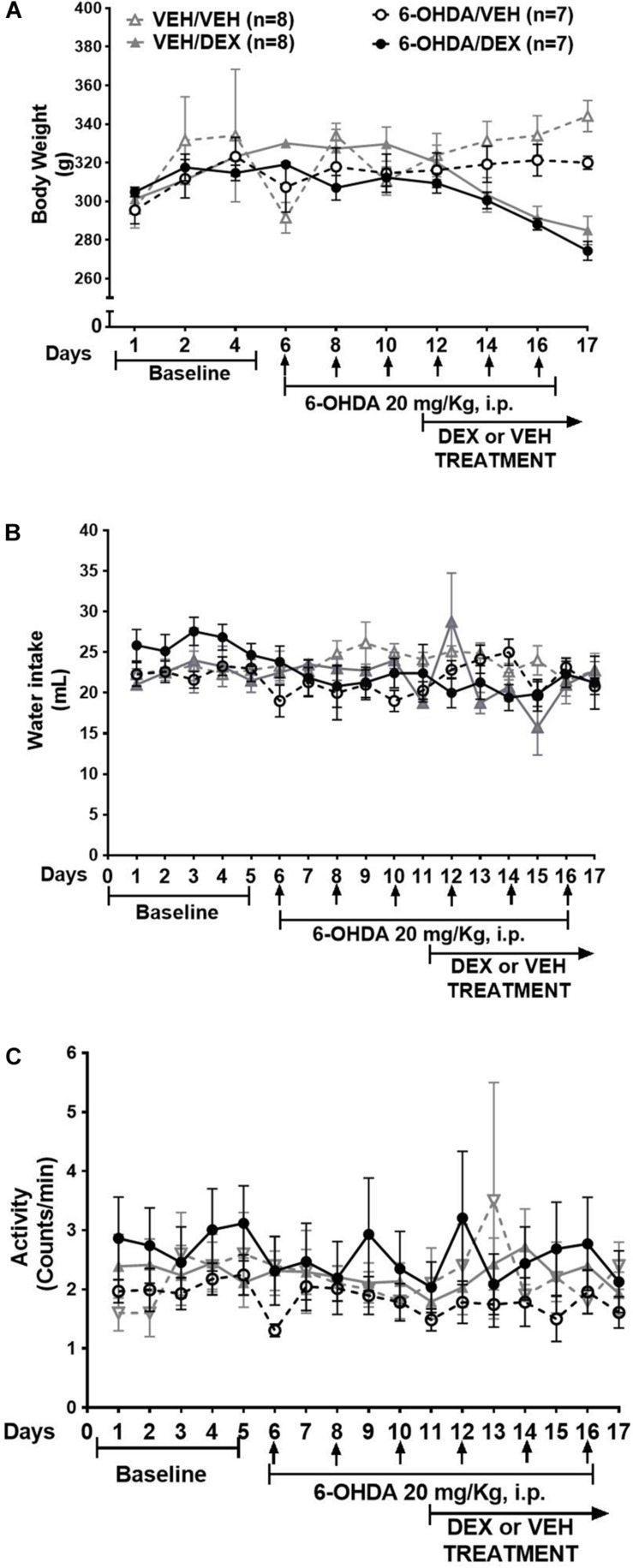
6-OHDA does not modify body weight, water intake, and activity. Data are shown as mean ± SEM of body weight **(A)**, water intake **(B)**, and activity **(C)** during 6-OHDA and DEX treatment. Statistical analysis was performed using two-way ANOVA with Tukey’s multiple comparison test.

In addition, body weight (Figure 4A) had a significant interaction between group and time (*p* = 0.0031) (Figure 4A). There was no significant difference between VEH/DEX and 6-OHDA/DEX groups. Water intake and activity did not change significantly after 6-OHDA or DEX administration (Figures 4B,C).

**FIGURE 5:**
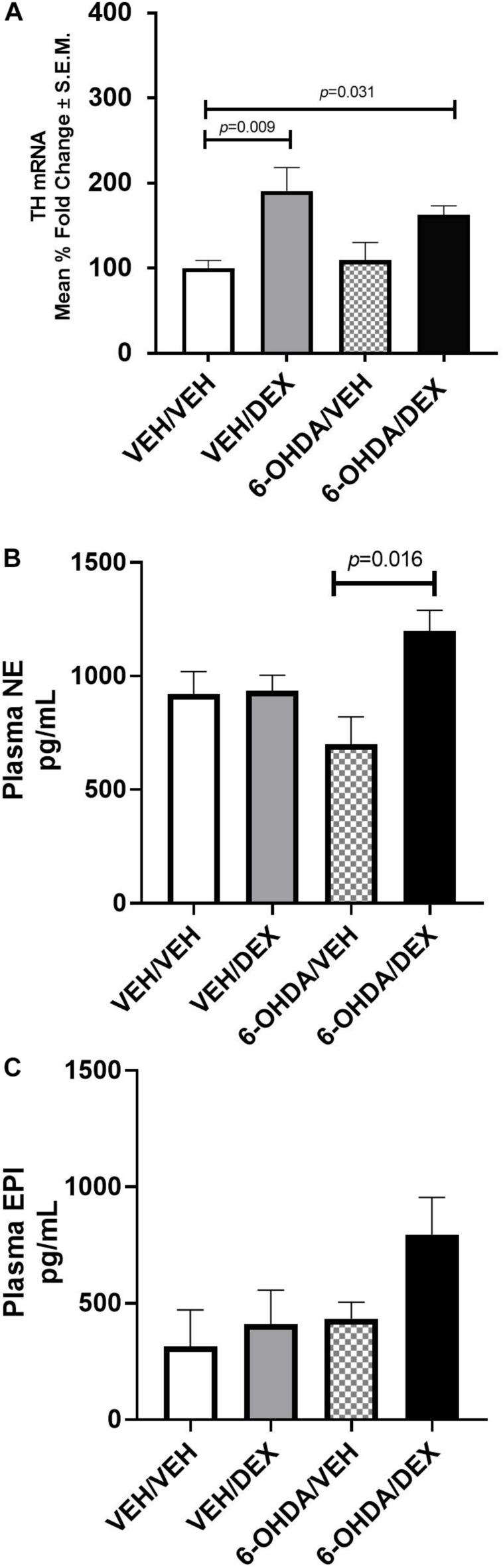
Effect of DEX and 6-OHDA treatment on TH mRNA **(A)**, plasma NE **(B)**, and EPI **(C)**. Data are presented as mean ± SEM. Kruskal–Wallis test and Dunn’s multiple comparison analysis were used to find differences between groups.

In order to identify the effect of DEX and 6-OHDA treatment on the catecholamine synthetic pathway, TH mRNA in the adrenal medulla and plasma concentrations of EPI and NE were measured. TH mRNA increased significantly in the VEH/DEX (*p* = 0.009 vs. VEH/VEH) and 6-OHDA/DEX (*p* = 0.031 vs. VEH/VEH) groups (Figure 5A). Plasma NE (Figure 5B) on the 6-OHDA/DEX group was higher than the one in the 6-OHDA-VEH group (*p* = 0.016). Concentrations of EPI (Figure 5C) were not significantly different among all groups (*p* = 0.072).

In the experiment with three injections of 6-OHDA before DEX (Supplementary Figure S1A), ΔMAP was different between treatment groups (χ^2^ = 20.6, *p* < 0.001). Moreover, ΔMAP in the VEH/DEX group was greater than the VEH/VEH (*p* < 0.001) and 6-OHDA/DEX (*p* = 0.008) groups. There was no significant difference (*p* = 0.120) in normalized ΔMAP between VEH/DEX and 6-OHDA/DEX groups (Supplementary Figure S1B). Regarding ΔHR, it was significantly different between groups (χ^2^ = 11.4, *p* = 0.009). In addition, ΔHR in the 6-OHDA/VEH group was higher than the VEH/DEX (*p* = 0.012) group (Supplementary Figure S1C). Normalized ΔHR (Supplementary Figure S1D) in the 6-OHDA/DEX group was lower than the VEH/DEX group (*p* = 0.002).

### Effect of the Combination of ADMX and 6-OHDA on DEX-Induced Hypertension

**FIGURE 6:**
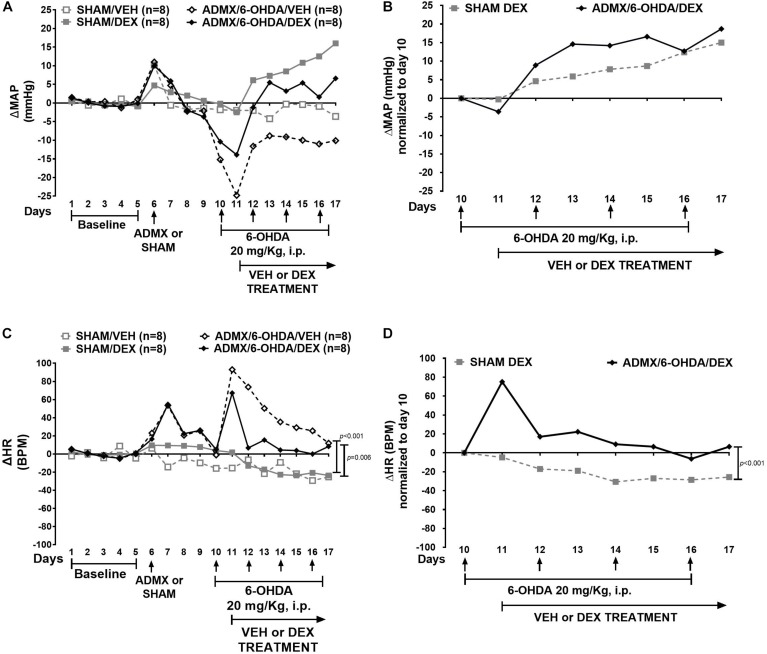
Effect of combined ADMX and 6-day 6-OHDA administration on ΔMAP and ΔHR in DEX-induced hypertension. Data represent the median of ΔMAP and ΔHR in animals treated with SHAM (gray) and ADMX/6-OHDA (black). Top panel depicts **(A)** effect of ADMX, 6-OHDA, and DEX on ΔMAP; **(B)** normalized ΔMAP to day 10. Bottom panel depicts **(C)** ΔHR and **(D)** normalized ΔHR to day 10. Comparisons were performed with Friedman Repeated Measures Analysis of Variance on Ranks with Tukey’s *post hoc* test. Confidence intervals are shown in Supplementary File S4.

In the experiment combining ADMX and 6-OHDA (Figure 6A), ΔMAP was not significantly different between groups (χ^2^ = 7.447, *p* = 0.059). Normalized ΔMAP (Figure 6B) was not different between SHAM/DEX and ADMX/6-OHDA/DEX groups (*p* = 0.161). ΔHR (Figure 6C) was different between groups (*p* < 0.001). ΔHR in the ADMX/6-OHDA/VEH was significantly higher than the SHAM/VEH (*p* < 0.001) and SHAM/DEX (*p* = 0.006) groups. Furthermore, it was lower in SHAM/VEH group (*p* < 0.001) than the ADMX/6-OHDA/DEX one. Normalized ΔHR (Figure 6D) in the ADMX/6-OHDA/DEX was higher than the one in the SHAM/DEX group (*p* < 0.001).

## Discussion

Hypertension is a disease of complex pathology that encompasses the sympathetic nervous system in addition to endocrine and renal mechanisms. The main finding in this study is that the pressor effect of DEX is resistant to surgical and chemical ablation of the peripheral sympathetic nervous system. In fact, the results showed that RDNX and ADMX do not reduce blood pressure response to DEX. Nonetheless, blood pressure upon DEX treatment decreases when animals had 6-OHDA and it does not exceed DEX pressor response, as seen in the RDNX and ADMX experiments.

### The Role of Renal Nerves in DEX-Induced Hypertension

Renal denervation resulting in a minimum of 50% reduction in NE content in the renal cortex is a therapeutic approach that is associated with the reduction of blood pressure (Wyss et al., 1987). In our study, the decrease in baseline MAP after RDNX was accompanied by a 98% decrease in renal cortex NE content. This indicates that there should be a decrease in sympathetic drive to kidney; reducing ion and water reabsorption. Furthermore, in the SHAM-RDNX group, MAP increased on the day when surgery was performed, and this was not seen in the SHAM-ADMX group; this difference could be because the anesthesia period in the second one was longer and might have caused this type of response, individual variability may be another factor contributing in this effect. That acute increase in MAP is not seen in the RDNX group probably due to the lack of renal nerves and compensatory mechanisms that are blocked upon the absence of renal tone. Contrary to that, the ADMX group shows an increase in MAP on that day, and they still have renal sympathetic nerves that could respond acutely to the reduction of adrenal catecholamines.

However, in this investigation, the absence of renal nerves did not prevent DEX-induced hypertension. These results are consistent with earlier reports where RDNX was not able to eliminate blood pressure elevations in the Dahl NaCl-sensitive rats (Wyss et al., 1987) and salt-sensitive-related hypertension (Foss et al., 2018; Tudorancea et al., 2018). Actually, RDNX exacerbates the response to DEX (Figure 1B); that may be associated with an increase in vascular reactivity. This is mediated by the presence of GR in endothelial and vascular smooth muscle; and includes several mechanisms that promote the elevation of blood pressure such as increased NE-induced vasoconstriction and vascular resistance, reduction of nitric oxide synthesis and cholinergic-induced vasodilation, COX activation, upregulation of angiotensin 1 receptors, and ion flux alterations (Goodwin et al., 2008). Moreover, GR knockout in vascular endothelial muscle reduces blood pressure in mice (Goodwin et al., 2011). Interestingly, these mice had circadian pattern alterations in blood pressure while being exposed to DEX. We did not observe alterations in the light/dark cycle of blood pressure and HR responses to DEX.

The role of renal nerves in vascular reactivity during DEX chronic exposure is not completely understood yet. RDNX can certainly reduce NE spillover, sympathetic activity, and consequently reduce hypertension; but its effects on vascular endothelial function are poorly studied. However, we found an elevated blood pressure response in animals with RDNX and DEX. This effect may be related to the time where RDNX was performed. For instance, in studies of deoxycorticosterone-salt hypertension, RDNX and ADMX were performed after the induction of hypertension (Lange et al., 1998; Jacob et al., 2005). We, on the other hand, performed RDNX and ADMX prior to DEX administration. Moreover, some central feedback mechanisms might have a small influence as well, for example, those involving renal afferents (Walkowska et al., 2013). Therefore, these factors may interact with vascular reactivity and oxidative stress generated by DEX, to increase blood pressure; nevertheless, their influence might not have the same impact level as these latter.

Another finding is that HR increases upon RDNX, but once DEX treatment starts, HR is lower in the RDNX group. This is consistent with clinical studies that show that RDNX reduces HR in hypertensive patients (Hering et al., 2013) and prevents cardiac remodeling produced by oxidative stress (Feng et al., 2017). This response most likely is driven by local cardiac responses to the absence of renal nerves and consequent modifications in renal function and water volume, maybe to maintain cardiac output. Contrary to this, HR reduction upon DEX treatment is not seen in vascular endothelial muscle-specific GR knockout mice (Goodwin et al., 2011). This suggests that in our study RDNX may enhanced GR effects on the heart. In addition, RDNX can also reduce cardiac renin–angiotensin system activation and preserve left ventricular ejection fraction (Sharp et al., 2018). Interestingly, the administration of minoxidil, a nitric oxide agonist increases cardiac output and reduces total peripheral resistance, but it does prevent DEX-induced hypertension either (Ong et al., 2009). Moreover, there is no correlation between DEX and hematocrit in that study. These hemodynamic variables were not measured in our investigation, and this may represent a limitation.

### Impact of ADMX on DEX-Induced Hypertension

Adrenal medullectomy does not reduce DEX pressor response, as seen in hypertension of renal origin (Finch and Leach, 1970a). The increase of blood pressure in the ADMX experiment after DEX administration is higher than the one in 6-OHDA and ADMX/6-OHDA experiments. This outcome could be the sum of different ongoing mechanisms such as: the activation of sympathetic vasomotor nerves (Kvetnansky et al., 1979) and vascular reactivity to catecholamines. For instance, renal sympathetic nerve activity may increase, as well as, NE spillover to the kidney, causing an elevation of Na^+^ and H_2_O reabsorption and blood volume augmentation. Furthermore, there are remnant levels of plasma EPI and NE that may act on adrenergic receptors, enhancing vasoconstriction. Vascular reactivity can involve adrenergic system alterations like impaired β2-mediated vasodilation (Bao et al., 2007; Berg et al., 2010), increased reactivity to dopamine (Abdulla et al., 2008), and NE via up-regulation of α2 receptors (Sripanidkulchai et al., 1987). However, a limitation of this study is the time when ADMX and DEX treatment were performed. It is probably that these mechanisms arise before DEX oral exposure and they synergize with GR actions in the vasculature, provoking the increase of blood pressure. Besides that, non-catecholaminergic mechanisms related to GC-induced hypertension could contribute to this response to DEX, for example: increased reactivity to arginine–vasopressin (Iijima and Malik, 1988), renin–angiotensin system activation (Sato et al., 1994); reduction of vasodilator gene expression such as cyclohydroxylase 1 and inducible nitric oxide synthase (Qiu and Baylis, 2000; Mitchell et al., 2003); and production of reactive oxidative species (Mondo et al., 2006). Therefore, the increase in blood pressure upon ADMX and DEX may be a convolution of multiple compensatory responses that are activated to maintain homeostasis in the body, and may provide a possible explanation for why the pressor response to DEX is resistant to any kind of sympathetic surgical ablation.

A caveat of this experiment is that plasma catecholamines were measured in paired-animals that were not implanted with telemeters and perhaps in those with the implant, the levels of catecholamines may not be the same. Particularly, Fischer 344 rats 3–4 months old show lower concentrations of plasma NE and EPI than older animals (Cizza et al., 1995). So, age may not affect plasma catecholamines during this study. The presence of plasma catecholamines upon ADMX indicates that there could be other sources of catecholamines such as sympathetic ganglia, nerve terminals, or lymphocytes (Sze and Hedrick, 1983; Cosentino et al., 2007). Therefore, sympathetic nerve activity and NE release might increase, producing an elevation in MAP during DEX treatment. That might also contribute to the elevation of HR in the ADMX/VEH group. Interestingly, after DEX treatment starts, HR decreases, suggesting there might be a reflex response to DEX-induced increases in blood pressure or a direct effect of DEX on the heart, but it is not changed by the medullectomy.

While ADMX is able to reduce blood pressure responses to mineralocorticoids, hypoxia, and some metabolic stressors (Shin et al., 1985; Lange et al., 1998), a similar response was not observed after synthetic GCs exposure. Historically, the adrenal medulla is considered an organ that responds to global stressors (Carter and Goldstein, 2015), but DEX chronic exposure may induce an allostatic load that is not reversible by solely removing the adrenal medulla. It seems there is a physiological adaptation to DEX, and one potential sign of that is that HR reduction upon DEX treatment is not modified in the ADMX/DEX group compared to the SHAM/DEX group in the normalized curves.

### Sympathectomy With 6-OHDA Prevents Augmentation of DEX Pressor Response

Increased vasomotor sympathetic nerve activity may be one compensatory mechanism that is triggered upon ADMX or RDNX. It is important to consider that plasma catecholamines may not always reflect systemic sympathetic nerve activity since there are anatomically specific sympathetic discharge and catecholamine reuptake mechanisms that behave differently (Esler, 2011). In addition, chronic sympathectomy can affect blood flow in different ways throughout the vasculature (Zhang et al., 1994). Therefore, in the final leg of our investigation, the role of peripheral sympathetic nerves in DEX-induced hypertension was studied using chemical sympathectomy with 6-OHDA. The first finding is that MAP in the 6-OHDA/DEX group is lower than the VEH/DEX group. Therefore, the loss of sympathetic nerves seems to reduce DEX pressor response. Despite this hypotensive effect of 6-OHDA, the pressor effect of DEX was not abolished as seen in the normalized curve. Similar findings have been reported in deoxycorticosterone-salt (Finch and Leach, 1970b), SHRs (Yamori et al., 1972), and bilateral renal clip hypertension models (Grewal and Kaul, 1971). Conversely, 6-OHDA completely prevents hypertension in the Dahl salt-sensitive rat (Takeshita et al., 1979) and in chronic intermittent hypoxia (Lesske et al., 1997). Indeed, none of those studies reported continuous recordings of baseline blood pressure nor made evident that the hypertension reduction might be due to a baseline decrease upon 6-OHDA exposure. In that sense, our animal model is the first one to show the characterization of systemic 6-OHDA effects on dynamic blood pressure recordings during baseline and chronic DEX treatment.

The pressor effect of DEX during 6-OHDA can be associated with compensatory mechanisms to sympathetic nerve destruction (De Champlain and Van Ameringen, 1972), as shown by an increase of TH activity in the adrenal medulla (Mueller et al., 1969) and preganglionic terminals (Sze and Hedrick, 1983). Evidence of that is revealed in our model as an elevation in TH mRNA and plasma NE in the 6-OHDA/DEX group. Moreover, NE decreased only 30% after six injections of 6-OHDA. Hence, this chronic scheme of 6-OHDA treatment creates a partial peripheral sympathectomy and it is possible that the other 70% of NE is released from remnant sympathetic fibers and the adrenal medulla that can be targets of DEX and GR genomic effects. TH is expressed in these sites (Burgi et al., 2011), and DEX could induce its transcriptional upregulation. In agreement with that, TH mRNA in the adrenal medulla increases in the VEH/DEX and 6-OHDA-DEX groups and it is consistent with the augmentation of plasma NE and MAP in the 6-OHDA/DEX group.

Nonetheless, catecholamines were not measured right after the first injection of 6-OHDA and toward the pre-DEX period. Despite that fact, baseline MAP was reduced until day 10 which may indicate there is a reduced sympathetic activity. Consequently, MAP returns to baseline and the preservation of DEX pressor response may be related to the activation of the catecholamine synthetic pathway in the adrenal medulla, the presence of nerve intact cell bodies, undestroyed sympathetic terminals upon 6-OHDA sympathectomy as well as nerve regeneration (Finch et al., 1973; Haeusler, 1984). Consistent with this, in the 3-day 6-OHDA experiment, MAP returns to baseline in the 6-OHDA/VEH group at the end of the telemetric recording and DEX pressor is maintained as well. Further experiments of nerve tracing and sympathetic nerve activity may be necessary to identify which fibers are active in this animal model of hypertension. The splanchnic nerve may be a potential source of sympathetic outflow (Zhen et al., 2019) in this type of hypertension as well.

Normalized ΔMAP curves between VEH- and 6-OHDA-treated animals are overlapped. Contrary to the curves seen in ADMX and RDNX, 6-OHDA administered during DEX oral treatment does not show the enhancement of this pressor response. Furthermore, the 3-day administration of 6-OHDA previous to DEX is not significantly increased from the VEH/DEX group. Therefore, sympathetic nerves may participate in the exacerbated pressor response of DEX after ADMX and RDNX. 6-OHDA might prevent a compensatory effect related to allostatic load to DEX. However, it is not enough to counteract GR actions to increase blood pressure. Perhaps, this response also includes the action of mineralocorticoids as seen in mice with GR haploinsufficiency (Ivy et al., 2018).

Furthermore, HR elevation in the 6-OHDA/VEH group may be the net result of ongoing responses to 6-OHDA-induced oxidative stress on the heart and the hypotensive state produced by the sympathectomy. 6-OHDA reduces cardiac NE for 5 days in rats and 3–14 days in mice (Joers et al., 2014). Therefore, this is consistent with HR elevation maintenance in the 6-OHDA/VEH group. However, after DEX oral treatment initiates, HR decreases in both groups although in the 6-OHDA/DEX group seems to have a lower level than the VEH group, probably due to loss of sympathetic fibers to the heart, cardiac GR signaling (Oakley et al., 2019), and an increase in central reflex mechanisms opposed to DEX-induced vascular reactivity.

The combination ADMX/6-OHDA shows a higher reduction in MAP baseline, but DEX pressor response was not eliminated. So, the loss of the adrenal medulla and the partial sympathectomy may enhance vascular reactivity to catecholamines and renal mechanisms associated with non-genomic effects of GR or mineralocorticoid receptors. In addition, normalized MAP in ADMX/6-OHDA/DEX group is closer to the SHAM group on days 16 and 17, as in the experiment with 6-OHDA alone for 6 days. Interestingly, αMPT attenuated the effect of DEX without modifications in baseline (Soto-Piña et al., 2016), suggesting that catecholamine synthesis from both the adrenal medulla and sympathetic nerves is inhibited simultaneously and this may prevent vascular reactivity and enhanced MAP responses during DEX treatment.

Finally, HR is not reduced during DEX treatment as seen in the other cases. The loss of adrenal catecholamines and sympathetic firing to the heart might alter signaling cascades associated with adrenergic receptors, acetylcholine pathways, or Ca^+2^, resulting in an enhanced tachycardia. Therefore, ADMX and 6-OHDA combination may counteract DEX effects on the heart. Indeed, 6-OHDA can modulate baroreflex sensitivity, reducing blood pressure and tachycardia in rats chronically treated with *N*-nitro-L-arginine methyl ester (Chaswal et al., 2015).

## Conclusion

In conclusion, hypertension induced by chronic DEX treatment is resistant to ablations of peripheral sympathetic nerves and the adrenal medulla. Furthermore, the adrenal medulla and renal nerves are essential for the regulation of baseline blood pressure, and also may play a role in the DEX-induced vascular reactivity in this animal model. Sympathetic nerves might play a role in the reactivity to DEX since MAP does not further increase upon chemical sympathectomy with 6-OHDA administered simultaneously to DEX.

## Data Availability Statement

The datasets generated for this study are available on request to the corresponding author.

## Ethics Statement

The animal study was reviewed and approved by the Institutional Animal Care and Use Committee (IACUC) of the University of Texas Health Science Center at San Antonio.

## Author Contributions

AS-P, RS, EF, CH-L, HG, and CR contributed in the research design, conducted the experiments, performed the data analysis, and wrote the manuscript. CF, HG, and CH-L participated especially in the radiotelemetric experiments and surgical procedures. CR assisted with qPCR methods and manuscript editing. EF participated in the tissue processing and HPLC analysis for catecholamines. EC-P and AB-A performed the statistical analysis and biological interpretation of the section “Results.”

## Conflict of Interest

The authors declare that the research was conducted in the absence of any commercial or financial relationships that could be construed as a potential conflict of interest.
