# Supplementary material for: Dexamethasone Causes Hypertension in Rats Even Under Chemical Blockade of Peripheral Sympathetic Nerves
Source: Front Neurosci. 2019 Dec 6;13:1305. doi: 10.3389/fnins.2019.01305 (PMC6909820; doi:10.3389/fnins.2019.01305)
Supplement: Supplementary file 1 [file Image_1.pdf]

Supplementary Figure 1

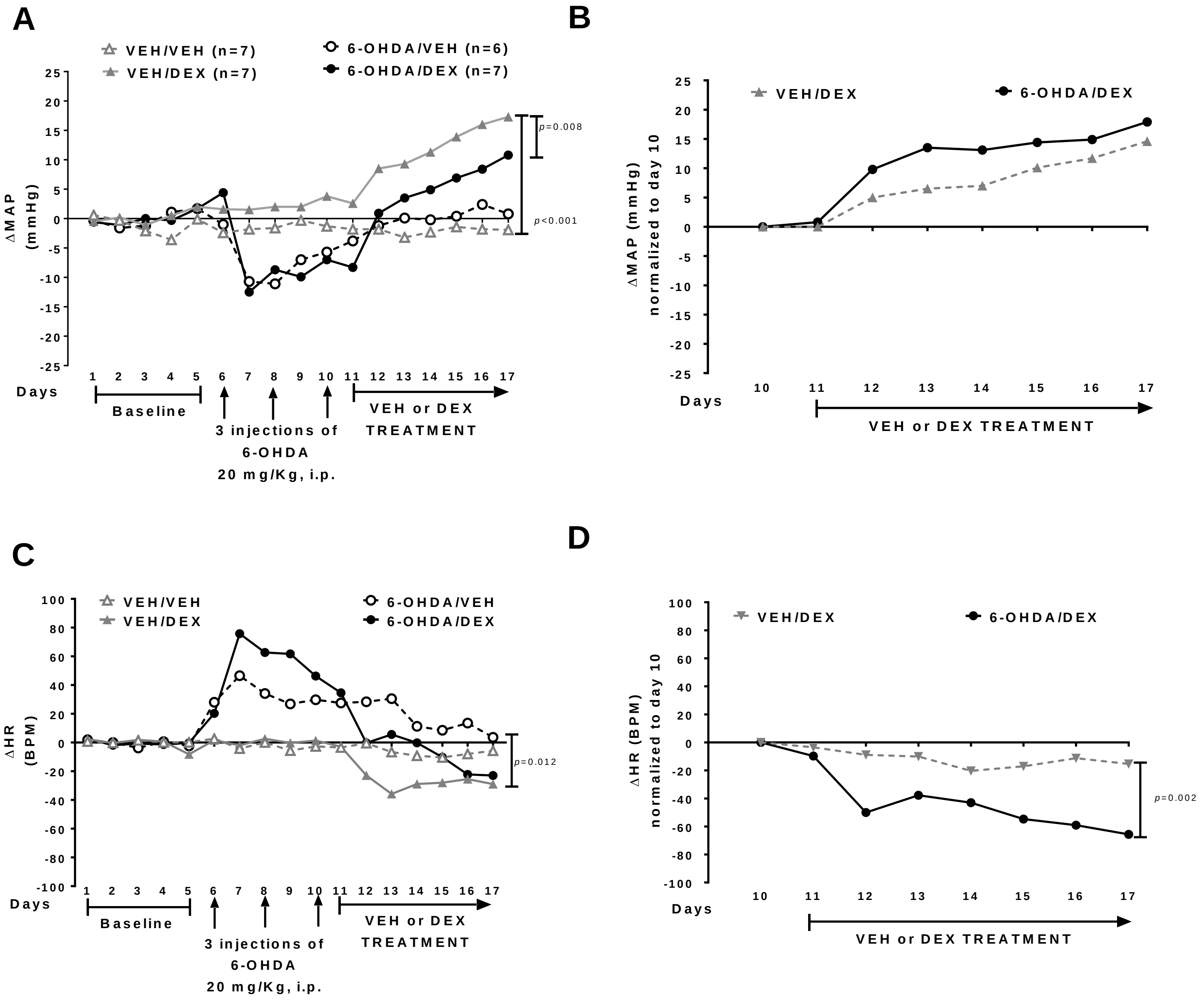

Fig. 1S: Effect of 3-day 6-OHDA administration on ΔMAP and ΔHR in DEX-induced hypertension. Data represent the Median and Upper and Lower limits of ΔMAP and ΔHR in animals treated with VEH (gray) and 6-OHDA (black). Top panel shows **(A)** Effect of 6-OHDA and DEX on ΔMAP and **(B)** ΔMAP normalized to day10. Bottom panel shows **(C)** ΔHR and **(D)** normalized ΔHR to day 10. Comparisons between treatment groups were performed with Friedman Repeated Measures Analysis of Variance on Ranks and Tukey *post hoc* test. Supplementary file 5 presents confidence intervals and median limits.
